# Candidate target genes for loss of heterozygosity on human chromosome 17q21

**DOI:** 10.1038/sj.bjc.6601848

**Published:** 2004-05-25

**Authors:** L DeMarchis, C Cropp, Z M Sheng, S Bargo, R Callahan

**Affiliations:** 1 Mammary Biology and Tumorigenesis Laboratory, National Cancer Institute, Bethesda, MD 20892, USA

**Keywords:** chromosome 17q21, loss of heterozygosity, plakoglobin

## Abstract

Loss of heterozygosity (LOH) on chromosome 17q21 has been detected in 30% of primary human breast tumours. The smallest common region deleted occurred in an interval between the D17S746 and D17S846 polymorphic sequences tagged sites that are located on two recombinant P1-bacteriophage clones of chromosome 17q21: 122F4 and 50H1, respectively. To identify the target gene for LOH, we defined a map of this chromosomal region. We found the following genes: *JUP*, *FK506BP10*, *SC65*, Gastrin (*GAS*) and *HAP1*. Of the genes that have been identified in this study, only *JUP* is located between D17S746 and D17S846. This was of interest since earlier studies have shown that JUP expression is altered in breast, lung and thyroid tumours as well as cell lines having LOH in chromosome 17q21. However, no mutations were detected in *JUP* using single-strand conformation polymorphism analysis of primary breast tumour DNAs having LOH at 17q21. We could find no evidence that the transcription promoter for *JUP* is methylated in tumour DNAs having LOH at 17q21. We suspect that the target gene for LOH in primary human breast tumours on chromosome 17q21 is either *JUP* and results in a haploinsufficiency for expression or may be an unidentified gene located in the interval between D17S846 and *JUP*.

We (Cropp *et al*, 1993; Cropp *et al*, 1994), and others (reviewed in (Bieche *et al*, 1997; Osborne and Hamshere, 2000), have shown that sporadic human breast carcinomas are characterised by the frequent loss of heterozygosity (LOH) on chromosome 17q12–21. A common speculation of these studies, based on Knudson's work (Knudson, 1971), is that LOH reveals a recessive somatic mutation in a gene, designated a tumour suppressor gene, within the other unaffected homologous chromosome. We have defined a region, using 17 polymorphic sequences tagged sites (STS), on human chromosome 17q21 that is affected by LOH in 30% of 130 sporadic breast tumours spanning the region between D17S746 to D17S846 (Cropp *et al*, 1994). Two P1-bacteriophage clones, 122F4 and 50H1, of human genomic DNA were identified that contain D17S746 and D17S846, respectively (Albertsen *et al*, 1994 and unpublished data). We have determined, partially, the nucleotide sequence of 122F4 and 50H1 as a strategy to identify candidate genes that could be targets for mutation in breast tumour DNAs having LOH in this region of chromosome 17q21. In the present communication, we identify six genes that are located on chromosome 17q21, their relative gene order and their transcriptional orientation. This study compliments the Draft Human Genome Nucleotide Sequence in that it physically locates the Plakoglobin (*JUP*) gene between the two polymorphic markers, D17S746 and D17S846, previously used to identify the smallest common region of LOH in sporadic breast cancer (Cropp *et al*, 1994). Analysis of breast tumour DNAs having LOH at 17q21 revealed no mutations in *JUP*, *SC65*, *FK506BP10* and *MGC20781* genes that are expressed in normal breast epithelium. *JUP* has also been screened for methylation of its transcription promoter regions to determine if an epigenetic mechanism could affect the expression of the remaining allele such as that found for *FHIT* in breast tumours (Yang *et al*, 2002). No methylation has been found in the series of 10 sporadic breast tumour DNAs having LOH at chromosome 17q12–q21.

## MATERIALS AND METHODS

### Characterisation of 17q12–21

Two P1 phages clones were used to analyse the 17q12–q21 region: 122F4 and 50H1 (generously provided by Dr Ray White, Department of Oncological Sciences, Huntsman Cancer Institute, University of Utah, Salt Lake City, UT, USA). Libraries of 122F4 and 50H1 were constructed using standard techniques with *Bam*HI or *SST*I genomic restriction fragments, respectively, cloned into the pBluescript II KS (+) vector (Stratagene, La Jolla, CA, USA) (Miyazaki *et al*, 1999). Subclones were subsequently ordered by size, restriction enzyme and nucleotide end-sequence analysis using universal primers from the vector. Nucleotide sequence analysis was performed using the ABI Big Dye Terminator sequencing kit (PE Applied Biosystems, Foster City, CA, USA) according to the manufacturer's instructions on an ABI 377 automated sequencer from PE Applied Biosystems (Foster City, CA, USA). The alignment of nucleotide sequences was determined with the Sequence Analysis Software Package by Genetics Computer Group (GCG), Inc. (Smithies *et al*, 1981). Within the GCG package fragment assembly was performed by the fragment assembly system based on the method of Staden (1980). Comparison of different sets of nucleotide sequences was analysed by the Best-Fit sequence alignment program (Needleman and Wunsch, 1970; Smith and Waterman, 1981). The genomic sequences obtained were compared using BLAST query with the GenBank. Colony hybridisation (Miyazaki *et al*, 1999) was used to identify additional clones of overlapping or adjacent restriction fragments that contained particular genes or exons of genes. Probes for colony hybridisation were cDNA clones for: the mouse homologue of *FK506BP10* (generously provided by Dr Stephanie L Simek, Science Applications International Corp., NCI-Frederick Cancer Research and Development Center, MD, USA) (Coss *et al*, 1995), human *JUP* (pHPG Ca 2.1, generously provided by Dr Werner W Franke, German Cancer Research Center, Heidelberg, Germany) (Franke *et al*, 1989) and human EST clones (Research Genetics, Huntsville, AL, USA) for *SC65* and *MGC20781*. Each probe was labelled with [*α*-^32^P]dCTP by random priming (Rediprime II kit, Amersham, Piscataway, NJ, USA).

### Reverse trancriptase–polymerase chain reaction (RT–PCR) analysis

For *JUP* expression, an RT–PCR assay was performed, using a Superscript One-Step RT-PCR kit with Platinum Taq Polymerase from Invitrogen (Carlsbad, CA, USA). Normal tissue total RNA (10 ng), purchased from Clontech, was used as template. The *JUP* primers for Exon 1A were: forward, 5′-CCGAGCTCAGTTCGCTGT-3′; reverse, 5′-TCGTTGAGC AGTTTGGTGAG-3′; for Exon 1B were: forward, 5′-ACCCGCT TTCCTGAAAGAAT-3′; reverse, 5′-AGCAGAAGCGAGACTG TCCT-3′. The GAPDH primers were used as a control for the amount of RNA tested: forward, 5′-CCCTTCATTGACCTCAACTAC-3′; reverse, 5′-CCACCTTCTTGATGTCATCAT-3′ to amplify a 600 base pair (bp) fragment. A measure of 25 *μ*l of 2 × mixture containing 0.4 mM of each dNTP, 2.4 mM MgSO_4_, 10 *μ*M of each primer, 1 *μ*l of Rt/Platinum Taq mix and autoclaved (RNAse-free) distilled water up to 50 *μ*l of total reaction volume. The cDNA synthesis and predenaturation was performed at 52°C for 30 min for one cycle and the PCR amplification was performed for 35 cycles at 94°C for 15 s, 52°C for 30 s and 72°C for 1 min. A final extension was performed at 72°C for 10 min.

### Mutation screening

‘Cold’ single-strand conformation polymorphism (SSCP) analysis was performed as described by Hongyo *et al* (1993) to identify mutations in *JUP* genomic from breast tumours having LOH on chromosome 17q21. The primers used correspond to intron sequences located 5′ and 3′ of the particular exon (Table 1

**Table 1:** Primers for the amplification of *JUP* exons

**Exon**	**Intron location^a^**	**Forward (5′–3′)**	**Intron location^b^**	**Reverse (5′–3′)**	**Gel running temp. (C°)^c^**	**Product (bp)^d^**
1	−16	ATACTCAGTAGCCACGAT	+13	CCCCATGCAATAGTCCC	15	254
2	−15	CTGCACTCAGTCCCTGTC	+6	ACCCTGCAGGGACCCTC	15	315
3	−1	CTGCCTTTGCCCTCCAG	+6	AACCCTGCTCACTGCATG	15	279
4	−6	GTACTAACCCCTGCCCAC	+13	TCAGGCCTCGGGAGAGTT	12	255
5	−4	CTCATTCCCTCTTTACCC	+3	CCCTCAAGGCCATCATACT	15	188
6	−8	TGATGGCACCCCTATCCC	+11	CAACCCCAGGCCCAGAT	15	157
7	−24	TTGGCTGCTCCCTGACTC	+22	CTGCTGCAGGGAGCTCCT	15	420
8	−9	ACTGCCCCTTTTTTTGCC	+175	GCATGGGACAGGTGCCTT	17–18	350
9	−22	AAGGCACCTGTCCCATGC	+53	TCTGGGACTCCTAACCTT	17–18	230
10	−12	TCCCCTGCTTCCCACGTC	+55	TTAAACGTCGGCCAGCC	12–13	249
11	−4	CCTGGCTTTTCCTTTCCTCTCT	+10	ACTACTGTGGTCCAACCT	15	178
12	−51	ACCAGCTGAGATCCAGCT	+16	TCTCCAGGGTCCTGAAGA	12–13	143
13	−20	TGCAGACCCTAGGACCGA	+43	GGAAAAGCCTCAAAGA	15	232

a The forward primer for Exon1 starts 16 bp 5′ to the translation start signal, for the other Exons the number given is the base pair separating the last base of the primer and the begining of the Exon.

b The number given is the base pair separating the last base of the Exon and the 3′ end of the reverse primer.

c The temperature at which the 4–20% polyacrylamide gel was run.

d The size of the PCR product is indicated base pairs.

). After an initial denaturation cycle of 94°C for 5 min, amplification from 100 ng genomic DNA was carried out with two cycles each of (94°C 10 min, 66°C 10 min, 72°C 20 min), (94°C 10 min, 64°C 10 min, 72°C 20 min), (94°C 10 min, 62°C 10 min, 72°C 20 min), (94°C 10 min, 60°C 10 min, 72°C 20 min) and then 25 cycles of (94°C 10 min, 56°C 10 min, 72°C 20 min) with a final extension cycle at 72°C for 7 min. All SSCP products were analysed on 4–20% precast polyacrylamide gels (Novex, San Diego, CA, USA) in TBE at 325 V for 60 min at a primer set-dependent temperature (see Table 1) using the manufacture's conditions. The gels were stained with SYBR Green II (Molecular Probes, Inc., Eugene, OR, USA) using the manufacture's conditions, visualised using a 340 nm UV viewing box and photographed.

### Methylation-specific PCR for the JUP transcription promoter

As a positive control for CpG methylation of genomic DNA, we have methylated a restriction fragment of recombinant genomic DNA containing the *JUP* transcription promoter. This was performed using the SssI methylase (CpG methylase) (New England Biolabs, Inc., Beverly, MA, USA) and the manufacturer's conditions. A PCR-based experimental protocol was used to detect hypermethylation of genomic DNA (Herman *et al*, 1996; Dracopoli *et al*, 1998). Briefly, 1 *μ*g of primary breast tumour genomic or control recombinant DNA containing the *JUP* promoter was treated with sodium bisulphite. Modified and nonmodified control and tumour DNA were used as templates in PCR reactions with FastStart Taq DNA Polymerase (Roche Molecular Biochemicals, Indianapolis, IN, USA). The reaction mixture contained 5 *μ*l of 10 × PCR buffer (containing 2 mM MgCl_2_), 1 *μ*l of 10 mM PCR Nucleotide Mix, 250 ng of each primer, 0.4 *μ*l FastStart Taq DNA Polymerase (2 U), 1 *μ*l of bisulphite modified DNA and distilled water to 50 *μ*l total volume. The primer pairs for unmethylated DNA: PgU F, forward 5′-TTGGAGTAGTTGTTGTTTGATTGT-GTT-3′; PgUR reverse 5′-CAAACCAAATCAAAATCAAACCAA-3′; and for methylated DNA: PgMeth F forward 5′-TAGTCGTCGTTCGATCGCGTC-3′; PgMethR reverse 5′-AACCGAATCGAAATCGAACCG-3′ have been previously described by Potter *et al* (2001) and lead to the amplification of 84 and 76 bp fragments, respectively. The PCR reactions were placed in an Applied Biosystems GeneAmp PCR System 9700 thermal cycler and activated at 95°C for 4 min, denatured at 95°C for 30 s, annealed at 56°C for 30 s and elongated at 72°C for 1 min for 35 cycles. A final extension of 72°C for 7 min and a 4°C indefinite hold completed the reaction. For analysis, 12 *μ*l of each PCR reaction was mixed with 5 × Hi-Density TBE sample Buffer (Invitrogen, Carlsbad, CA, USA) and loaded onto a 6% TBE Gel (Invitrogen). The gel was run at 200 V for 30 min, stained with ethidium bromide and visualised on a UV light box.

## RESULTS AND DISCUSSION

**Figure 1:**
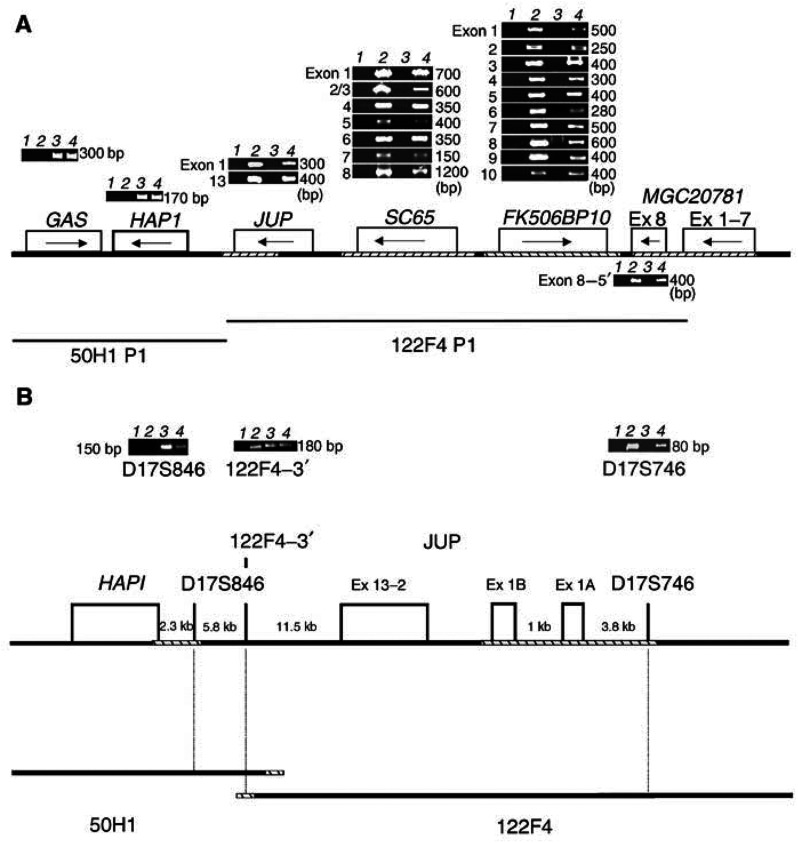
(**A**) A partial map of human chromosome 17q12–q21. The PCR products of an analysis of the P1-phage clones are shown for the presence of exons corresponding to the indicated genes. The arabic numbers represent the templates used to perform the PCR amplification of the indicated exon: lane 1, water control; lane 2, 122F4; lane 3, 50H1 P1-phage clones; lane 4, genomic DNA control. The intron/exon primers of all the genes are available upon request. The sizes of the PCR-amplified fragments are indicated in bp. The arrow indicates transcriptional orientation 5′–3′ of the indicated genes. The portions of the map with right-hand hatch marks correspond to regions in which the nucleotide sequence of the exon/intron junctions was determined. The regions of the map in which the entire nucleotide sequence was determined are indicated with left-hand hatch marks. The centromeric end of the map is on the left and the telomeric end is on the right side. (**B**) A map of the region of chromosome 17q12–q21 affected by LOH. The positions of Exons 1A, 1B, the remainder of *JUP* exons and *HAP1* as well as D17S846, 122F4-3′ and D17S746 on the chromosome map are indicated. The PCR products of an analysis of the P1-phage clones, 50H1 and 122F4, are shown for the presence of D17S846, HUM122F4-3′ (accession number L32940) and D17S746. The arabic numbers represent the templates used to perform the PCR amplification of the indicated exon: lane 1, water control; lane 2, 122F4; lane 3, 50H1 P1-phage clones; lane 4, genomic DNA control. The regions of the map in which the entire nucleotide sequence was determined are indicated with left-hand hatch marks.

To identify new genes with a potential tumour suppressor function and determine their position within the region of chromosome 17q12–21 affected by LOH, we have determined the nucleotide sequence of portions of the 122F4 and 50H1 P1-phage clones of human genomic DNA that span the region between the D17S846 (centromeric) and D17S746 (telomeric) polymorphic markers (Figure 1

). Nucleotide sequence analysis of *Bam*HI and *SST*I fragments of 122F4 demonstrated the presence of the *JUP* gene. Previous studies (Aberle *et al*, 1995; Whittock *et al*, 2000) localised JUP within this area of chromosome 17. In the present study, we can now physically link *JUP* to *SC65* (Exons 1–8), *FK506BP10* (Exons 1–10) and *MGC20781* (Exon 8). *FK506BP10* is a member of a family of genes that encode proteins that are immunophilins that bind FK506 and rifampysin and possess prolyl:prolyl isomerase activity (Siekierka *et al*, 1989; Standaert *et al*, 1990). The normal function of *Fk506BP10*-*, SC65*- and *MGC20781*-encoded proteins is not known. Only the last exon (Exon 8) of *MGC20781* is located on 122F4. The results of a PCR analysis of the exons for each of these genes on 122F4 are shown in Figure 1A.

We have also localised by PCR analysis D17S746 and D17S846 on these P1-phage clones (Figure 1B). D17S846 is located ∼7.8 kb from one end of the recombinant genomic DNA in P1-phage 50H1 and is 2291 bp from the transcription promoter region of *HAP1*. The D17S746 locus is present on 122F4 but not on 50H1, suggesting that it is located near *JUP*. This observation was confirmed by nucleotide sequence analysis of a recombinant clone from a library of SSTI-digested 122F4 DNA. D17S746 is located 3865 bp telomeric of *JUP* Exon 1A (Figure 1B). The HUM122F4-3′ STS locus is located 191 bp from one end of the genomic fragment in the 122F4 P1-phage clone.

The transcriptional orientation of the genes could be deduced by PCR and nucleotide sequence analysis. Thus of the genes on 122F4, *JUP* Exon 13 is located ∼11.5 kb from the HUM122F4-3′ STS locus (Figure 1). This is consistent with the direction of transcription being from the telomeric to centromeric regions of the chromosome. This analysis was also anchored with the observation that only Exon 8 of *MGC20781* was found on 122F4 and is consistent with the transcriptional orientation of this gene being in the same direction as *JUP* (Figure 1). In addition, Exon 8 of *MGC20781* could be linked to a 3.4 kb PCR fragment containing Exon 10 of *FK506BP10*, which is consistent with the transcriptional orientation of *FK506BP10* being in the opposite direction as *JUP*. Similarly, Exon 8 of *SC65* and D17S746 were linked in an 11 kb PCR fragment (Figure 1) consistent with the transcriptional orientation of *SC65* as being in the same direction as *JUP*. These results confirm the Human Genome Map and physically locate the STS loci HUM122F4-3′ and D17S746, respectively.

As genes were identified in this study, we screened genomic DNA from 11 primary human tumours having LOH in this region of chromosome 17 by nucleotide sequence analysis. No missense, nonsense or frameshift mutations were detected in *SC65*, *FK506BP10* or *MGC20781* in these tumours DNAs. Gastrin (*GAS*) and *HAP1* were not tested since they are not expressed in the mammary gland. When it was clear that among the genes that were analysed, only *JUP* was located between D17S746 and D17S846; *JUP* genomic DNA was tested by the more sensitive ‘cold’ SSCP analysis (Osborne *et al*, 1991; Hongyo *et al*, 1993) for mutations, none were found (data not shown).

**Figure 2:**
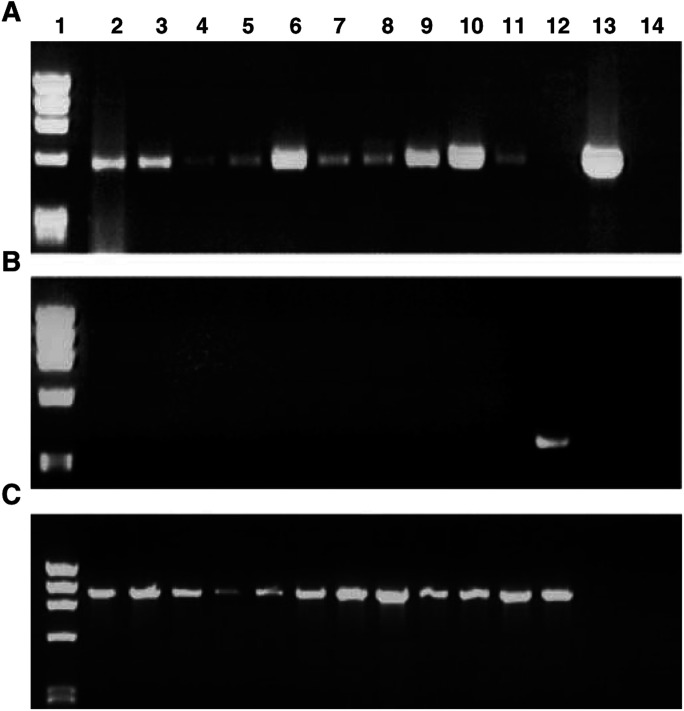
An RT–PCR assay of *JUP* Exon 1A (**A**), Exon 1B (**B**) and GAPDH (**C**) RNA expression. Lane 1 is Marker *Hae*III-digested phi X174 DNA. RT–PCR was performed on RNA from: lane 2, heart; lane 3, kidney; lane 4, peripheral blood lymphocytes; lane 5, liver; lane 6, placenta; lane 7, lung; lane 8, muscle; lane 9, ovary; lane 10, mammary gland; lane 11, small intestine; lane 12, EST-395811 cDNA containing Exon 1B; lane 13, pHPG Ca 2.1 cDNA containing Exon 1A; lane 14, water.

*JUP* has two potential transcription promoters. *JUP*, Exon 1A was expressed in 10 tissues tested (Figure 2

), whereas Exon 1B could not be detected in any of these tissues (Figure 2). However, five ESTs that contain Exon 1B from a placenta cDNA library (NIH_MGC_21) have been reported in the GenBank. One of these is the positive control shown in Figure 2B (lane 12).

**Figure 3:**
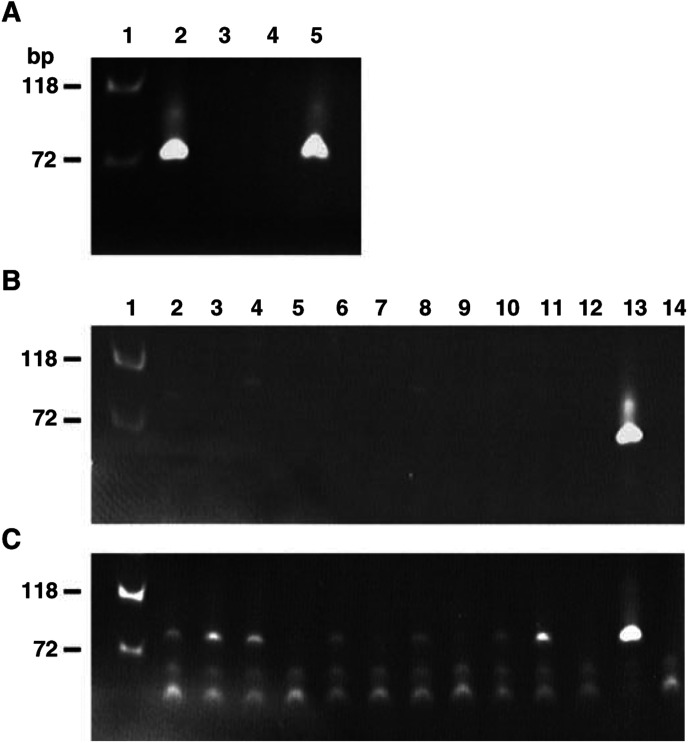
Methylation-specific PCR of the *JUP* promoter region in primary breast tumour DNAs having LOH on chromosome 17q21. (**A**) Test of primer pairs for PCR of unmethylated and *in vitro* methylated recombinant genomic DNA containing the *JUP* promoter region: lane 1, Marker *Hae*III-digested phi X174 DNA; lane 2, methylated-specifc primers and methylated *JUP* genomic DNA; lane 3, methylated specifc primers and unmethylated *JUP* genomic DNA; lane 4, unmethylated specifc primers and methylated *JUP* genomic DNA; lane 5, unmethylated specifc primers and unmethylated *JUP* genomic DNA. PCR analysis of primary breast tumour DNAs with methylation specific primers (**B**) or primers specific for unmethylated *JUP* genomic DNA (**C**). The DNAs were: lane1, Marker *Hae*III-digested phi X174 DNA; lane 2, tumour 16; lane 3, tumour 20; lane 4, tumour 26; lane 5, tumour30; lane 6, tumour 44; lane 7, tumour 62; lane 8, tumour 63; lane 9, tumour 89; lane 10, tumour 117; lane 11, tumour127; lane 12, unrelated genomic DNA; lane 13, control methylated recombinant *JUP* promoter region (**B**) and control unmethylated recombinant *JUP* promoter region (C); lane 14, water control.

Winn *et al* (2002) demonstrated that plakoglobin expression is reduced or absent in a subset of human lung cancers. Further they showed that re-expression of plakoglobin inhibits transformed cell growth, suggesting *JUP* is a tumour suppressor gene. Blanco *et al* (2002) found that 76% (13 out of 17) invasive ductal carcinomas of the breast had reduced levels of plakoglobin. Potter *et al* (2001) have shown that in some thyroid tumours and cell lines, which express low or undetectable levels of plakoglobin, the *JUP* promoter is hypermethylated. To see if a similar phenomenon affects the remaining *JUP* allele in breast tumours having LOH at chromosome 17q21, we have examined the methylation status of the transcription promoter region for *JUP*. The specificity of the primers for methylated and unmethylated *JUP* Exon 1A is shown in Figure 3A

. No PCR product (Figure 3B, lanes 2–12) the size of the control fragment (lane 13) of methylated *JUP* Exon 1A was detected using the tumour DNAs as the template. However, as shown in Figure 3C, lanes 2–12, PCR products of the same size as the control (lane 13) for unmethylated DNA was detected with tumour DNA templates. *JUP* Exon 1B was not tested for its methylation status since it is not active in the mammary gland (Figure 2).

In conclusion, we have focused on 122F4 and 50H1 P1-phage clones because they contain (Albertsen *et al*, 1994, and unpublished data) overlapping genomic DNA fragments that spanned a region of chromosome 17q21 affected by LOH. Our nucleotide sequence analysis of the ends of the genomic DNA in these P1-phage clones is consistent with an overlap of 2.3 kb. At the present time, this region of chromosome 17q12–q21 is ambiguous with respect to the presence and location of particular genes identified in the Human Genome Project. For instance, D17S746 has not been physically mapped relative to *JUP* in the Human Genome Project map. However, consistent with our data, Aberle *et al* (1995), have identified three recombinant cosmid clones of this region of human chromosome 17q21 that contained both D17S846 and *JUP*. It is possible, therefore, that the target gene for LOH on chromosome 17q21, in primary human breast tumours, could be located in the intervening nucleotide sequences between D17S846 and *JUP* (17 489 bp, GenBank). At the present time, however, there is no evidence for a gene in this region of chromosome 17q21 in the Human Genome Project map.

Another possibility is that *JUP* is the target for LOH. *JUP* encodes *γ*-catenin or plakglobin and is highly homologous to *β*-catenin (Gumbiner, 1996). Both are components of cell–cell adherens junctions linking cadherin receptors to the actin cytoskeleton (Ben-Ze'ev and Geiger, 1998). In addition, plakoglobin is a component of desmosomes (Franke *et al*, 1989; Ben-Ze'ev and Geiger, 1998). Immunohistochemical analysis of primary human breast tumours for the expression of the catenin family demonstrated that if one of these proteins is downregulated, the function of the others in suppressing metastasis is altered (Bukholm *et al*, 1998; Gonzalez *et al*, 1999). In another study, it was shown that expression of plakoglobin was selectively downregulated in metastatic tumours (Bukholm *et al*, 2000). We have found no evidence that in tumours having LOH between D17S846 and D17S746 the remaining allele of *JUP* is mutated. Nor have we found evidence that the transcription promoter of *JUP* is methylated preventing transcription of the remaining allele in these tumours. Therefore, we speculate that haploinsufficiency of *JUP* caused by LOH is sufficient to contribute to breast tumour progression. A future study specifically aimed at correlating LOH of *JUP* and reduced plakoglobin levels in primary breast tumours seems warranted.
