# Candidate target genes for loss of heterozygosity on human chromosome 17q21

**DOI:** 10.1038/sj.bjc.6602117

**Published:** 2004-08-24

**Authors:** L DeMarchis, C Cropp, Z M Sheng, S Bargo, R Callahan

**Correction to:** *British Journal of Cancer* (2004) **90**, 2384–2389. doi:10.1038/sj.bjc.6601848

Owing to a typesetting error, the first author's name in the above article was shown incorrectly. The correct author listing is:

L De Marchis, C Cropp, ZM Sheng, S Bargo and R Callahan

